# Mercury Exposure and Health Effects in Indigenous People from the Brazilian Amazon—Literature-Scoping Review

**DOI:** 10.3390/ijerph22081159

**Published:** 2025-07-22

**Authors:** Maria da Conceição Nascimento Pinheiro, Fabiana Costa Cardoso, Leonardo Breno do Nascimento de Aviz, José Aglair Barbosa de Freitas Junior, Márcia Cristina Freitas da Silva, Margareth Tavares Silva, Dirce Nascimento Pinheiro, Saul Rassy Carneiro, Elaine Rodrigues Pinheiro, Tereza Cristina Oliveira Corvelo

**Affiliations:** 1 Programa de Pós-Graduação em Doenças Tropicais, Núcleo de Medicina Tropical da, Universidade Federal do Pará, Belém 66075-110, Brazil; mconci@ufpa.br (M.d.C.N.P.); fabianacostacardoso2@gmail.com (F.C.C.); freitasjunior19@hotmail.com (J.A.B.d.F.J.); 2 Instituto de Ciências Biológicas, Universidade Federal do Pará (UFPA), Belém 66075-110, Brazil; marciaf@ufpa.br (M.C.F.d.S.); margarethfarmaceutica@gmail.com (M.T.S.); 3 Faculdade de Enfermagem, Instituto de Ciências da Saúde, Belém 66005-240, Brazil; dircenp@ufpa.br; 4 Faculdade de Medicina, Instituto de Ciências da Saúde, Belém 66005-240, Brazil; saul@ufpa.br; 5 Department Faculdade de Medicina, Centro Universitário Metropolitano da Amazônia, Belém 66053-000, Brazil; elaine_rodrigues215@hotmail.com; 6 Laboratório de Imunogenética, Instituto de Ciências Biológicas, Universidade Federal do Pará (UFPA), Belém 66075-110, Brazil; tereza@ufpa.br

**Keywords:** indigenous, Amazon, mercury, exposure, prevalence

## Abstract

Background and purpose: Indigenous people in the Brazilian Amazon are exposed to mercury by eating methylmercury-contaminated fish. The lack of information on the health effects of prolonged exposure to mercury hinders the implementation of mitigation programs offered by the Brazilian government. This article aims to evaluate the studies that have investigated mercury exposure in indigenous people living in the Brazilian Amazon. Methods: A scoping review of the literature was conducted from studies published between 1995 and 2024 in Portuguese, English, and Spanish that evaluated mercury (Hg) concentrations in hair samples in indigenous people from the Brazilian Amazon. Results: Using total mercury (TotalHg) values in hair samples, we analyzed exposure levels, prevalence, and toxic effects. We found 15 epidemiological studies with a cross-sectional design and sample sizes ranging from 31 to 910 participants. Four studies involved children and mothers, four of which were associated with clinical outcomes and three of which analyzed genetic polymorphism. Most of the communities evaluated had a high prevalence of mercury exposure, showing levels ranging from 0.8 to 83.89 µg/g, and the highest average TotalHg concentration was found among the Kayabi. Mercury was associated with hypertension, cognitive disorders, worse mental health indicators and central and peripheral neurological disorders. Conclusions: It is concluded that indigenous people in the Brazilian Amazon experience exposure levels that are causing damage to their health, and control measures must be adopted to prevent the situation from worsening.

## 1. Background

Brazil has a great diversity of indigenous communities, which are concentrated in the Amazon region. Most of these populations face poor sanitary conditions, difficulties in accessing public education and health services, and attacks by infectious agents responsible for endemics and epidemics [1]. In addition to these factors, they are also subjected to prolonged exposure to heavy metals, especially mercury, as they are located in areas where artisanal gold-mining activity prevails, which uses large quantities of elemental mercury. These conditions place indigenous people in social-vulnerability conditions [2].

Various studies involving traditional Amazonian populations, including riverine and indigenous people who have traditionally consumed a fish-based diet [3,4,5,6,7], have demonstrated mercury levels in hair samples above the reference limit of 6 µg/g established by the World Health Organization (1990) [8]. Additionally, studies like Silva and Lima (2020) [9]; Souza-Araujo et al. (2016) [10]; and Martín-Doimeadios et al. (2014) [11] involving Amazon fish, frequently consumed by these populations, also revealed that some species, especially predators, showed mercury levels above the reference limit for human consumption, as established by the World Health Organization (2008) [12].

Few studies have evaluated mercury exposure in indigenous populations, and, among these, some have investigated the prevalence of exposure [3,5,13,14,15]. Others have evaluated the effects associated with this exposure [5,7,14]. The process of bioaccumulation of mercury in fish from areas close to mining sites occurs when the metal enters the aquatic ecosystem in its inorganic form, which can be transformed into the organic form, when enzymatically complexed by bacteria to methyl radicals, which results in methylmercury [16]. In its most toxic form, methylmercury is rapidly assimilated by living organisms and accumulates in aquatic food chains [17].

Acute exposure to this element can result in a wide range of adverse effects, including abdominal cramps, nausea, vomiting, diarrhea, cough, chest pain, cyanosis, tachypnea, lethargy or agitation, and intestinal mucosal corrosion and irritation. Additionally, respiratory distress, gastrointestinal hemorrhage, and pulmonary edema may occur [18]. Conversely, when prolonged exposure occurs, the kidneys, nervous system, and digestive system are most affected by the effects of the substance in question [19,20].

Article 21 of the Declaration on the Rights of Indigenous Peoples—DDPI [21]—approved in 2006, states that indigenous peoples have the right, without discrimination, to the improvement of their economic and social conditions, including education, employment, sanitation, health and social security, and others. However, indigenous people’s situation in the Amazon requires efforts to implement public policies that can change the current reality and thus fulfill the recommendations of the DDPI. The lack of information on the health problems caused by prolonged exposure to mercury among indigenous people in the Brazilian Amazon makes it difficult to implement the mitigation programs offered by the Brazilian government. In order to contribute to public policies on indigenous health, with a view on controlling exposure and preventing the occurrence of contamination by methylmercury, this study aims to evaluate the studies that have investigated exposure to mercury in indigenous people living in Brazilian territory, particularly those in the Brazilian Amazon.

## 2. Material and Methods

This study presents a scoping study or scoping review of the literature conducted in accordance with the criteria established by the Preferred Reporting Items for Systematic Reviews and Meta-Analyses (PRISMA).

The research question was defined based on the levels of exposure to mercury through the measurement of total mercury (TotalHg) in hair samples, which is a biomarker indicative of prolonged exposure to mercury via food or consumption of food contaminated by methylmercury. The acronym PCC (population, control, and context) was used to describe the study design. Consequently, the research question was formulated as follows: “Is the indigenous population of the Brazilian Amazon being affected by non-occupational exposure to mercury?”

### 2.1. Search Strategy and Information Sources

The searches were conducted between January 2023 and April 2024 in three electronic databases: PubMed, Virtual Health Library (VHL), and Scielo. The searches were conducted in Portuguese, English, and Spanish. The keywords used were described using the search terms Medical Subject Headings (MeSH) and Health Sciences Descriptors (DeCS). The descriptors researched were identified using the PCC strategy: (mercury or methylmercury) and exposure and (indians or amerindian people or indigenous) and (amazon or brazil) and hair, using descriptor AND and OR as connective).

### 2.2. Eligibility Criteria

Original observational studies were included in the analysis, which evaluated exposure to mercury through measurements of total mercury (TotalHg) and/or methylmercury (MeHg) in hair samples. The studies considered prevalence, exposure levels, and effects associated with exposure in the indigenous population of the Brazilian Amazon. This study included both adult and child participants of both sexes. The selected outcomes were as follows: prevalence, exposure levels, and effects of exposure.

All original studies, published between 1995 and 2024, were included in the analysis. In order to ensure the quality and reliability of the data, literature reviews, theses, and dissertations, as well as studies that involved exposure to inorganic mercury, whether occupational or non-occupational, were excluded.

### 2.3. Data Selection and Extraction Process

Two experienced reviewers (MCNP and JABFJ) conducted the initial search and selection to identify the titles and abstracts of potentially relevant studies. Each abstract was evaluated by the two reviewers independently. If at least one reviewer deemed a reference to be eligible, the article was obtained in its entirety. The two authors then proceeded to analyze the articles independently in order to select those that would be included in the review. In the event of a discrepancy, the authors reached a consensus to resolve the issue. Manual citation tracking was also conducted on the selected articles.

It should be noted that the scope review does not establish the assessment of the methodological quality of the studies as an essential criterion. Consequently, no quality assessment was carried out.

**Figure 1:**
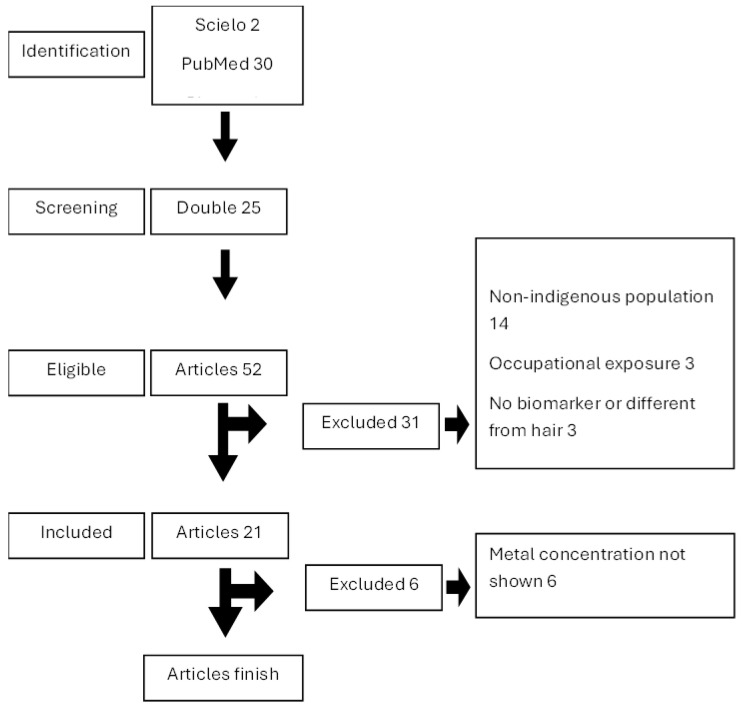
Flowchart of the article selection process.

A total of 77 studies were found, 2 in Scielo journals, 30 in the PubMed database, and 45 in Bireme; of these, twenty-five were duplicates. From the 52 eligible articles, 31 were excluded because the articles did not meet the eligibility criteria, such as those involving non-indigenous populations (14), involving occupational exposure (3), absence of a biomarker of exposure or different from hair (3), outside the Brazilian Amazon (8), literature review studies (2), and other issues of interest or outside the scope of the topic (1). From this process, 21 original clinical–epidemiological studies involving indigenous people from the Brazilian Amazon remained. However, after the complete reading of these studies, some did not show mercury concentrations in hair samples (6) (Figure 1).

The risk of bias was assessed individually for the selected studies using the Joanna Briggs Institute (JBI) critical appraisal checklist. The risk classification consisted of high, moderate, and low, thus excluding those with high publication.

This review has limitations, given that as it encompasses articles with varying methodologies, including differences in sample size and selection methods, we cannot guarantee the uniformity of data collection, but it does present a consistent analysis of the results achieved by each study.

## 3. Results

**Figure 2:**
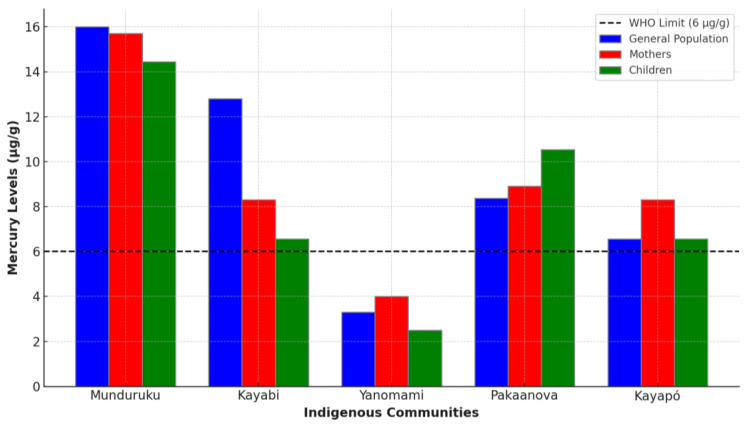
Comparison of mercury levels in indigenous Amazonian populations.

Prolonged exposure to mercury from consuming contaminated food is the most common form observed in riverine and indigenous communities in the Brazilian Amazon and is associated with the frequency and intensity of fish consumption in the diet [22]. By cultural tradition, these communities depend on fish for their survival. Hair is used as a bioindicator of this form of exposure to estimate levels of total mercury (TotalHg) and/or methylmercury (MeHg). In this review, we analyze the mercury exposure levels through the values measured in hair samples and the prevalence and effects of this exposure in different indigenous people living in the Brazilian Amazon. The results show that a significant proportion of samples exceed the WHO safety limit of 6 µg/g, particularly in communities closer to areas of intense artisanal mining activity (Figure 2).

We found fifteen original articles published between 1995 and 2024. From these, seven (46.7%) were carried out exclusively on Munduruku (MK) indigenous people, two (13.3%) on Munduruku and Kayabi, one (6.7%) on a Kayapó tribe, two (13.3%) in the Pakaánova village, and three (20%) on a Yanomami tribe. All had a cross-sectional design and sample sizes ranging from 13 to 910 participants. Four studies involved children and mothers, four were associated with clinical outcomes, and three analyzed genetic polymorphism. The Munduruku ethnic group has been the most studied. In two studies, the MK were compared to the Kayabi Indians [23,24], and in seven studies, the MK people were included individually (Table 1) [3,5,7,13,25].

### 3.1. Effects of Mercury on Adults

Chronic exposure to mercury, particularly methylmercury, has been linked to a range of health concerns in adults, with a particular emphasis on neurological implications. Studies on indigenous populations in the Amazon have demonstrated that adults who have been exposed present with motor deficits, such as ataxia, as well as cognitive abnormalities, including memory loss and reduced verbal fluency [7]. Furthermore, elevated mercury levels have been linked to sensory disturbances, including paresthesia and alterations in visual field perception. These impairments have the potential to significantly impact the quality of life and functional capacity of affected individuals [5]. Of particular concern are the neurotoxic effects of mercury in communities that rely on rivers contaminated by artisanal gold mining for their livelihoods, including fishing and agriculture.

### 3.2. Effects of Mercury on Mothers and Children

Mothers and children are specific groups that are particularly vulnerable to mercury toxicity. In women, accumulated mercury can cross the blood–brain barrier and the placental barrier, increasing the risk of neurological damage to both mother and fetus. Children are even more vulnerable to the neurotoxic effects of methylmercury due to their stage of development. Studies in indigenous populations show that exposed children have learning difficulties, delays in neuromotor development, and low intelligence quotient (IQ) scores, with some showing significant deficits in memory and cognitive abilities [14,30].

### 3.3. Maternal–Fetal Risks

The maternal–fetal risks of mercury exposure are well documented. During pregnancy, mercury can cross the placenta and accumulate in developing fetal tissues, causing irreversible damage to the central nervous system. Studies show that fetuses exposed to mercury have an increased risk of developing congenital malformations, neurological deficits, intrauterine growth retardation, and even fetal death. In addition, mothers who consume mercury-contaminated fish during pregnancy may not only jeopardize their own health but also the health of the newborn, who may have permanent neurological abnormalities. Exposure to mercury early in life, including during pregnancy, is a major cause of long-term cognitive and motor deficits [7,30].

### 3.4. Risk of Bias

To guarantee the methodological rigor of the included studies, the JBI checklist was employed to evaluate the potential for bias in cross-sectional studies. This instrument permitted the assessment of elements such as participant selection, exposure measures, the control of confounding factors, and the statistical analysis of the data. The rigor of the statistical analysis was reviewed to ensure that the methods applied were appropriate for the study design and data type, thereby enhancing the credibility of the results (Table 2).

## 4. Discussion

### 4.1. Mercury Exposure

Mercury exposure can cause a wide range of symptoms, both acute and chronic, which vary in adults, children, and fetuses (Table 3). Hair is a commonly used biomarker for assessing mercury exposure in the general population and prenatal exposure. It is considered a non-invasive collection method and is a highly useful matrix for carrying out a temporal analysis of exposure, since hair grows continuously, allowing for the evaluation of different time periods and seasonality [12,32]. According to the WHO (1990) [8], the maximum TotalHg values in hair samples for individuals with high fish consumption should not exceed 6 µg/g.

Human hair has been regarded as an optimal biosample for mercury analysis due to a number of factors. One of the key advantages of this method is that mercury can remain in keratinized material for extended periods due to its incorporation into the keratinized structure of the material in question during hair growth. This process occurs due to the composition of the hair, which is formed by different layers that offer protection to its interior. The majority of hair is composed of proteins (65–95%), water (15–35%), and lipids (1–9%), which contribute to the permanence of the element in the sample without significant issues. This allows for the detection of a wide range of concentrations, provides distinct data on recent or chronic exposure related to certain elements, and is a non-invasive method that is easy to obtain and does not present major concerns about transportation and storage. In general, toxic substances can be incorporated into the hair matrix by two primary means: absorption from the external environment and circulation through the bloodstream. The latter process carries bioaccumulative materials to the hair strands through passive diffusion [33,34].

Mercury can come from a variety of sources, including soil, water, air, and food. Exposure can occur in different ways in humans, with women of childbearing age, pregnant women, and children being more susceptible to the harmful effects of contamination [35]. Placental transfer is associated with the greatest adverse health effects in the affected population, as women ultimately transfer mercury (Hg) to the fetus when consuming foods containing Hg [36]. The main route of exposure to Hg in the population is the consumption of contaminated food. The dynamics of mercury in aquatic ecosystems is complex and influenced by many factors, including soil type, river flow, fish age, season, pH, productivity of aquatic ecosystems, local methylation characteristics, among others. The degree of contamination of fish is largely dependent on their position in the food chain, size and age [37]. Herbivorous fish (tambaqui, jaraqui, pirapitinga, pacu) have lower levels of contamination than carnivorous fish (dourada, filhote, piranha, tucunaré, and hake) [37,38].

In addition to the consumption of contaminated fish, which is the main source of mercury exposure for indigenous people in the Amazon, there are other important pathways that contribute to the risk of contamination. Artisanal gold mining (garimpo) is one of the main sources of mercury release into the environment. During the extraction process, mercury is used to separate the gold and is then burned, releasing toxic vapors into the atmosphere. Mercury is also released into rivers where aquatic microorganisms convert it into methylmercury, a highly toxic form. In addition, daily activities such as bathing and water collection expose these populations to mercury in contaminated waters, even if direct ingestion of the water is not the primary route of exposure [5,39].

Mercury deposited in soil can contaminate people through direct contact or accidental ingestion of particles, particularly in areas where mining is intensive. These multiple exposure pathways highlight the complexity of the problem, which requires an integrated approach that considers environmental and public health risks to protect indigenous populations exposed to mercury [7,14].

Levels of exposure to mercury in Munduruku villages have been variable, as shown by the studies that assessed exposure in Sawré Muybu’s general population, the Poxo Muybu and Sawré Aboy tribes. The results of five of these studies indicated exposure levels that are capable of causing damage to the health of this population. In all the villages studied, the concentrations exceeded the tolerance limit established by the WHO (2008) [12]; however, the Sawré Aboy had the highest concentrations, reaching a maximum of 23.9 µg/g [3,5,7,29]. These villages are located in the Tapajós region where gold mining has been promoting large-scale changes in land use in the traditional territories of the Amazon, causing direct and indirect socio-environmental impacts on local populations, including damage to food security, the local economy, people’s health, and the different ecosystems [3,5].

In the indigenous MK people of Sai Cinza Village, the highest concentrations were found in children aged 7 to 12 years, whose average concentration of Total Hg was 14.45 µg/g and, in mothers aged 14 to 44 years, 15.70 µg/g [28]. The results show that maternal exposure among MKs is much higher than exposure to non-indigenous riverine women in the Amazon, as demonstrated by [40].

When assessing the epidemiological risk of vertical transmission of mercury, they found an average of 3.65 µg/g in a study involving mothers in vulnerable situations in the state of Acre, in a border area in the Pan-Amazonian region [37]. The study by Corvelo et al. (2014) [41] also assessed mercury exposure in women of reproductive age, in the Tapajós region, where in 1999, the proportion of volunteers with mercury levels > 10 µg/g was approximately 68%. In general, exposure to mercury decreased among women of reproductive age, but the potential risks to reproduction and human health were still an issue, as 22% of the woman continued showing high mercury levels > 10 µg/g in 2012.

In the Tapajós region, in the state of Pará, exposure among the indigenous Kayabi people was assessed in order to compare cardiovascular risk parameters to MKs [23]. In this study, the average concentration of TotalHg in Kayabi hair was 12.8 µg/g, and in MK hair, it was 3.4 µg/g. In contrast, fish consumption by the Kayabi was also higher than that of the MK indigenous people, corroborating the findings of other studies which recognize the role of the frequency and quantity of fish ingestion on mercury exposure levels [22].

The TotalHg values presented by the indigenous MK and Kayabi people were higher than the exposure levels found in Amazonian riverside dwellers, whose dietary habit is also the frequent consumption of fish [22,42]. When compared to the exposure levels of Colombian Indigenous (34.9 ± 2.4 µg/g), it was found that the Kayabi are less exposed than other indigenous groups in the Pan-Amazon region [43]. These authors aimed to assess exposure in the Yaigajé Apaporis National Natural Park in the Colombian Amazon, specifically within the communities of Bocas de Taraira, Ñumi, Vista Hermosa, and Bocas de Uga, and found groups with average concentrations ranging from 23.0 ± 1.2 μg/g to 34.9 ± 2.4 μg/g.

More than two decades ago, mercury exposure levels in indigenous Kayapó people were assessed. In the Kikretum community (state of Pará), 251 indigenous people were investigated, whose children showed median values of 6.55 µg/g of TotalHg, ranging from 2.0 to 20.4 µg/g, and among mothers, a median concentration of 8.30 µg/g was found, ranging from 0.8 to 13.1 µg /g, with 25% of them showing levels higher than 10 µg/g, which indicates the possibility of maternal–fetal risk due to exposure to methylmercury [44].

In Rondônia state, nine hundred and ten (910) indigenous people from various villages of the Pakaánova ethnic group were investigated on mercury exposure levels. The average concentration in the general population was 8.37 µg/g and ranged from 0.52 to 83.89 µg/g. Among children under the age of two, it was 10.54 µg, and in those aged 3 to 5, it was 9.34 µg, while in mothers, the average level was 8.91 µg/g [27]. In these indigenous communities, both maternal and child exposure showed higher levels than the MK who inhabit the Tapajós region in the state of Pará, where artisanal gold mining prevails. In all age groups of the Pakaanovas, the average levels of TotalHg exceeded the safety limit established by national and international standards for populations that ingest fish frequently.

Among the indigenous groups investigated, the prevalence of exposure to mercury, TotalHg greater than 6 µg/g hair [12], was higher in the Yanomami, where the majority (92.3%) of the tribe’s general population had levels above reference values [15]. However, Brazilian indigenous people from other ethnicities studied showed a predominance of more than 50% of exposed individuals, but with mean and median levels of TotalHg higher than those found in the Yanomami. In the study by Achatz et al. (2021) [5], the prevalence of exposure to MeHg was higher than that found in the indigenous Yanomani people, being 22.8 µg/g, as also shown in the work of Basta et al. (2021) [3] and Perini et al. (2021) [14], which presented mercury values of 23.9 µg/g. This prevalence should arouse the concern of public health authorities to create strategies to prevent exposure and control the toxic effects of mercury in these people.

### 4.2. Genetics

Genetic alterations associated with mercury in indigenous people were investigated in three studies. The study by Klautau-Guimarães et al. (2005) [24] evaluated individual differences in susceptibility to MeHg and its relationship with the polymorphism of the detoxifying enzyme, glutathione S-transferase (GSTs). The study was conducted in two different villages (MK and Kayabi). The highest concentrations of TotalHg were found among the Kayabi-indigenous people, who had a 26 percent null genotype frequency for GSTM1 (0/0), while in the MK indigenous people, the levels were lower and the null genotype frequency for GSTM1 was 0 percent. No association was found with other markers studied, such as hemoglobin, haptoglobin, and glucose 6-phosphate dehydrogenase [24]. The general Kayabi population had an average TotalHg concentration of 14.75 ± 1.0 µg/g, and the MKs had an average concentration ranging from 3.65 ± 1.88 to 4.26 ± 2.16 µg/g. They also found that 62.2% of the Kayabi sample population had an average mercury concentration above 10 μg/g, and 26% of these showed the null genotype for the GSTM 1 enzyme.

GST polymorphism was also investigated in a non-indigenous population (riverbank dwellers) from the Tapajós region by Barcelos et al. (2012) [45]. In this study, the authors found an average TotalHg level of 10.4 ± 7.4 µg/g, which was associated with GSTM1 polymorphism. The results obtained in indigenous and non-indigenous populations highlight the protective role of GSTM against mercury damage [24,45].

More recently, Silva et al. (2023) [29] investigated the association of the GSTP1 A > G polymorphism with TotalHg levels and neurological signs possibly caused by prolonged exposure to mercury in three villages of the Munduruku people (Sawré Muybu, Poxo Muybu, and Sawré Aboy) involving 107 adult indigenous individuals. An association was found between the polymorphism of the enzyme and signs of neurotoxicity induced by methylmercury. These findings were interpreted by the higher frequency of the GSTP1 AA genotype associated with higher MeHg concentrations in residents of the Sawré Aboy village compared to those of the other two villages studied (Sawré Muybu and Poxo Muybu).

Silva et al. (2023) [29] associated AA genotypes with methylmercury genotoxicity in the Munduruku-indigenous people, and particularly in the Sawré Aboy villa, a higher frequency of AA and Ag alleles was found together with the highest concentrations of TotalHg and MeHg in hair and blood, associated with a higher risk of neuropathies with somatosensory and cognitive disorders. In addition, the GG allele was associated with a protective effect, causing the natives to show low levels of MeHg. These results contradict those of Medina-Perez et al. (2021) [46] and Gundacker et al., 2009 [47], who consider that GG is the genotype with the greatest risk for the effects of mercury exposure. These contradictory results may be related to the size and method of sample selection.

APOE4 is another risk marker for high levels of mercury exposure. Arrifano et al. (2018) [48] investigated ancestry and susceptibility to possible damage caused by mercury in two groups of riverside populations in the Amazon (Tapajós and Tucuruí regions) and found that both ancestry and susceptibility to mercury toxicity were associated with the presence of APOE4 and the indigenous origin of the genetic markers in the population studied. They also showed that fifty-nine of the individuals evaluated were at maximum risk of toxic effects exhibiting TotalHg levels greater than 10 µg/g, and the presence of APOE4 was considerably above the safe limits according to WHO (2008) [12].

In the study by Perini et al. (2021) [14], the genetic polymorphism of the enzyme delta aminolevulinic acid dehydratase (ALAD) was investigated in indigenous children. This enzyme is important for the synthesis of heme and plays an important role in the toxicokinetics of metals, especially the transport of metals throughout the body. Perini et al. (2021) [14] identified two children from the Sawré Muybu village with levels above 6 µg/g of mercury in their hair. These children present neurological symptoms such as changes in the visual field, memory impairment, distal neuropathy, and finger amyotrophy. The association of genetic alteration with high levels of mercury is mainly due to the fact that the alteration promoted in the amino acid by the polymorphism in ALAD results in a negatively charged isoenzyme, which has great affinity for inorganic metals and, therefore, higher levels of this in the organism [49].

### 4.3. Effects on Child Growth

Among the studies that evaluated exposure in indigenous child populations, such as Kayapó [26], Munduruku SaiCinza [25], Pakaanova [28], and Yanomami [30], indigenous Munduruku children from Sai Cinza showed higher exposure levels (greater than 14 µg/g), suggesting the possibility of risks to neurodevelopment, which were observed in the population at levels equal to or greater than 10 µg/g [5,7]. A more worrying scenario was observed in indigenous children, of which 18.8 percent presented motor changes at MeHg levels above 6 µg/g, and in this group, 62 percent of mothers had exposure levels greater than 6 µg/g [13]. The conditions of social vulnerability of indigenous peoples in the Brazilian Amazon put maternal and child health at risk, exposing riverside and indigenous children in this region to the risks of exposure to mercury, which requires more consistent government action.

In 2024, in the study carried out by Jacques et al. (2024) [30], in the indigenous Yanomami land, in the Brazilian Amazon forest, state of Roraima, a group of 117 children were evaluated for levels of environmental contamination and neurodevelopment. Among the subjects evaluated, TotalHg concentrations ranged from 0.16 to 10.20 µg/g, with an average of 3.3 µg/g; 79.4% had levels above 2 µg/g, and there was no significant difference between the sexes. In 58 children, it was possible to estimate the total intelligence quotient (ITQ), and its result was associated with mercury levels, in which a low score on the test was associated with a 5 times risk of having high mercury levels in hair samples, as well as identifying that these results were more significant among children with a high consumption of fish. In a study involving riverside children living in communities close to the Madeira River, Rondônia, it was also observed that high concentrations of TotalHg in hair samples were linked to lower performance in neuropsychological function tests, the Wechsler Abbreviated Scale of Intelligence—WASI, the same one used in the aforementioned study [23].

Indigenous MK women of lactating age (12–49 years) and their respective children were evaluated for exposure levels and outcomes related to child growth and development [13]; 62% of women and 18.8% of children exceeded the reference limit of 6 µg/g. The median MeHg level was 7.115 µg/g, and the highest concentrations were recorded in the Sawré Aboy community: 12.67 µg/g. These levels of exposure may be associated with the increased frequency of fish consumption by indigenous peoples.

Considering the safe reference levels of 6 µg/g for women of reproductive age, high levels of mercury were also found in riverside women in the state of Rondônia, in the Brazilian Amazon, considering the safe reference levels of 6 µg/g for women of reproductive age [8], especially in riverside and rural communities, whose levels ranged from 1.02 a 146.80 µg/g. Bello et al. (2023) [50], in a five-year cohort study involving different communities (riverside, rural, mining areas and urban areas), evaluated exposure in pregnant women, whose TotalHg concentrations in hair were generally considerably above safe limits according to the standards of WHO, probably due to the high consumption of contaminated fish [12].

However, in this study, it was possible to observe significant differences between the groups investigated, since women from the mineria areas, most of the time immigrants from other regions of Brazil, as a result of their diet including few weekly meals with fish, had mercury levels within of ideals. Furthermore, this study observed the relationship between the moment of pregnancy, the postpartum period, and capillary mercury levels and, when comparing the findings, observed a decrease in metal levels six months after birth in all groups evaluated, which reinforces findings from studies such as that by [7].

Maternal exposure to mercury through fish intake increases the risk of negative pregnancy outcomes, particularly for the fetus, and the effects depend on the quantity, frequency of intake, and species consumed, as well as the protective factors present in this diet, such as presence of selenium [51]. Selenium is a mineral that acts in oxidation and antioxidant reactions, which is important in the mercury-detoxification process. The Hg/Se correlation has been investigated in indigenous and non-indigenous people. In a study involving indigenous Wari-Pakaanovas, a 1:1 ratio of TotalHg and Se was demonstrated in hair samples, suggesting a protective role against the toxic action of mercury [27]. This relationship was also demonstrated in non-indigenous, pregnant, riverside women in the Amazon [51].

### 4.4. Effects on Blood Pressure (BP)

Blood pressure outcomes were assessed in two studies [3,23]. Two studies published in 2005 and 2021 involved the MK (Tapajós) and Kayabi (Madeira) groups. In addition to mercury exposure levels, Dórea et al. (2005) [23] assessed the effects on erythrocytes, BMI, and blood pressure. TotalHg levels in the three MK villages ranged from 2.5 ± 1.4 to 6.0 ± 2.9 µg/g. They found no association with the parameters assessed. Among the 47 indigenous individuals assessed, the average TotalHg concentration was 12.8 ± 7.0 µg/g, which was higher than that of the indigenous MK people. The difference between the two groups studied can be explained by the greater frequency of fish consumption among the Kayabi Indians than the MK [23]. The authors demonstrated a trend indicating decreasing blood pressure with increasing age among individuals with the highest fish consumption (Kayabi). They found no significant difference between the BMIs of the tribes, and no signs compatible with mercury poisoning were detected in the clinical assessment [23].

In 2019, exposure levels were determined in three Munduruku villages in the Tapajós region to investigate the association with hypertension. Among 193 indigenous people assessed, TotalHg concentrations ranged from 1.4 to 23.9 µg/g, exhibiting a significant difference between the villages studied. The prevalence of exposure, considering levels equal to or greater than 6 µg/g, was 57.9 percent, showing higher levels in the Sawré Aboy village. The association with increased blood pressure was recorded in indigenous Munduruku women with TotalHg levels equal to or greater than 6µ/g [3]. In a study involving indigenous Canadians, a higher prevalence of hypertension was observed in the population with higher levels of mercury exposure [52]. In Brazil, particularly in non-indigenous populations that consume fish in the Amazon, an association was found between systolic arterial hypertension and levels of TotalHg above 10 µg/g [53].

The role of heavy metals in the genesis of hypertension is probably due to oxidative stress and inflammation, which promote endothelial or renal dysfunction and enzymatic disturbances related to the mercury-detoxification process [54]. High levels of hypertension are a risk factor for cardiovascular disease, and the organic form of methylmercury has been associated in a dose–response manner with cardiovascular outcomes [55].

Arterial obstruction is one of the most commonly reported problems in current studies on mercury. The hypothesis is that mercury binds to the S-adenosylmethionine sulfhydryl group and inactivates an enzyme that acts as a necessary cofactor for catecholamine-o-methyltransferase, an enzyme required to convert norepinephrine, epinephrine and dopamine by methoxylation. This results in a syndrome reminiscent of a pheochromocytoma crisis, leading to malignant hypertension in acute mercury toxicity and significantly increasing urinary catecholamines in chronic mercury toxicity. This can aid in the diagnosis of mercury-induced hypertension [56].

### 4.5. Effects on Mental Health and the Central Nervous System

In a study published in 2021, Achatz et al. discussed the impact of illegal gold mining on the well-being of the Munduruku community and explored the possible relationship between chronic exposure to MeHg and worsening mental health in three villages located in the middle Tapajós. A total of 109 indigenous people aged between 12 and 72 years, distributed between the Sawré Muybu, Poxo Muybu, and Sawré Aboy villages, were assessed. The median concentration of TotalHg was 7.4 (2.0–22.8 µg/g). From these, 28 percent had MeHg levels equal to or greater than 10 µg/g, and 70 percent had values equal to or greater than 6.0 µg/g and scored a trend towards the worst mental health indicators associated with MeHg levels equal to or greater than 10 µg/g.

Oliveira et al. (2021) [7] assessed the impacts of chronic exposure to MeHg on the somatosensory, motor, and cognitive systems in 110 indigenous people over the age of 12 in the three MK villages mentioned in the study by Achatz et al. (2021) [5] and concluded that these indigenous individuals are twice as likely to experience cognitive deficits and alterations in verbal influence when MeHg concentrations are greater than 10 µg/g [7].

Studies on the indigenous Yanomami population in the Amazon in 2024 stood out, as levels above safe mercury limits were demonstrated in different biological markers [15,57]. In the study by [31], which included 154 Yanomami-indigenous individuals from the upper Mucajaí River, all of whom had some level of mercury, ranging from 3.9 ± 1.7 μg/g, 30.3% some peripheral neuropathy, and 34.8% reduced cognitive performance, which is closely attributed to the metal’s biomagnification capacity in the aquatic trophic chain and its ability to overcome the blood–brain and placental barriers. Karri et al. (2016) [58] and Santos-Sacramento et al. (2021) [59] addressed the neurotoxicity of heavy metals, such as mercury, on human health and highlight that the level of risk of both cognitive and neurological impairment will depend, to a large extent, on its intensity and metal-biochemical interactions in the brain.

Studies on a non-indigenous population in the Tapajós region, demonstrating TotalHg concentrations similar to those of indigenous people in this region, showed a low prevalence of neurological alterations that suggested mercury intoxication [60], and when submitted to psychophysical tests to investigate somatosensory disorders, they found no association with TotalHg concentrations when compared to the control group [61]. However, the authors discuss the role of mercury associated with other socio-environmental factors in the occurrence of the neurosensory alterations found [61].

According to the WHO [8] and Harada (2005) [62], concentrations of TotalHg above 50 µg/g can be associated with alterations in the central nervous system. However, evidence shows that these manifestations can appear at lower levels of exposure to MeHg, as demonstrated in indigenous people in Canada [63].

The experience of the Minamata accident in Japan revealed that somatosensory disorder was the first neurological manifestation and the most frequent disorder in acute MeHg poisoning [62]. Furthermore, it is a common manifestation in neuropathies of various causes. However, neurological manifestations, even in different study groups, take into account the intensity and time of exposure to the contaminant, a fact that highlights the differences between studies in the Brazilian Amazon and in Minamata. Therefore, there is a need to promote new, original studies involving indigenous people, with priority on research that can establish a causal link.

## 5. Limitations

Although this study makes an important contribution to understanding the impact of mercury exposure on indigenous populations in the Amazon, some limitations should be considered when interpreting the results. First, the cross-sectional nature of the study prevents the establishment of causal relationships between mercury exposure and the observed health outcomes. Longitudinal studies would be needed to determine more precisely how chronic mercury exposure evolves over time and its long-term effects. In addition, sampling was limited to certain communities, which may not fully reflect the diversity of experiences and levels of exposure in other regions of the Amazon.

## 6. Conclusions

The scientific literature involving prolonged exposure to mercury in indigenous people of the Brazilian Amazon is scarce; only 14 studies were found from 1998 to 2023. All of the studies found were cross-sectional observational studies, and most used non-probabilistic samples. The prevalence of mercury exposure with levels greater than 6 µg/g was high in all communities, especially the Ianomami. TotalHg concentrations ranged from 0.8 µg/g to 85.89 µg/g. Associations were observed between concentrations of TotalHg and/or MeHg and increased BP in women, showing MeHg levels above 10 µg/g representing twice the chance of cognitive deficit and verbal impairment in those over 12 years old. Also, MeHg concentrations greater than or equal to 10 µg/g were associated with worse mental health indicators, delayed gross motor development, anemia, and central and peripheral neurological disorders. New primary studies are needed to better assess mercury exposure in the indigenous populations of the Brazilian Amazon.

## Figures and Tables

**Table 1:** Non-occupational mercury exposure among indigenous populations in the brazilian amazon (1998–2023).

Author/Year	Location/ Indigenos/n	Hg (µg/g) Children	Hg (µg/g) Mother	Hg (µg/g) General Population	Percentage of Individuals Exposed	Clinical Outcome
Barbosa A. C. et al., 1998 [26]	(Pará) Kayapó (Kikretum): 251	6.55 (2.0–20.4)	8.30 (0.8–13.1)	NI	25% > 10 µg/g	NI
Campos M. S. et al., 2002 [27]	(Rondônia)Wari Pacaa Novos: 13	NI	NI	12.1 ± 0.15	NI	NI
Santos E. C. O. et al., 2002 [25]	Munduruku Sai Cinza: 320	X: 14.45 (7–12 anos)	X: 15.70 (14–44 anos)	X: 16 ± 18.9	NI	NI
Santos E. C. O. et al., 2003 [28]	(Rondônia) Pakaanova/910	X: 10.54 (<2 anos) X: 9.34 3–5 anos	X: 8.91	X: 8.37 (0.52–83.89)	NI	NI
Dórea J. G. et al., 2005 [23]	(Pará) 3 vilas Munduruku (Rondônia) Kayabi: 47	NI	NI	X: 2.3 ± 1.4–6.0 ± 2.9 X: 12.8 ± 7.0	NI	NI
Klautau-Guimarães M. N. et al., 2005 [24]	(Pará) Munduruku/117 (Rondônia) Kayabi/65	NI	NI	3.65 ± 1.88– 4.26 ± 2.16 11.97 ± 6.82– 17.86 ± 9.82	NI	0% GSTM1 null 26% GSTM1 null
Vega C. M. et al., 2018 [15]	(Rondônia) Yanomami/239	NI	NI	0.4–22.1	92.3% Hg ≥ 6	NI
Basta P. C. et al., 2021 [3]	(Pará) Munduruku (3 villages)/197	NI	NI	1.4–23.9	57.9 Hg ≥ 6	Association with hypertension in women
Oliveira R. A. A. et al., 2021 [7]	(Pará) Munduruku (3 villages)/110	NI	NI	XMeHg: 8.7 ± 4.5 7.4 (2.0–22.8)	NI	MeHg > 10 µg/g A two-fold increase in the likelihood of cognitive deficits and verbal impairment was observed in individuals aged 12 and older.
Achatz R. W. et al., 2021 [5]	(Pará) Munduruku (3 villages)/109	NI	NI	MeHg: 7.4 (2.0–22.8)	70% MeHg ≥ 6 28% MeHg ≥ 10	MeHg ≥ 10 Associated with worse mental health indicators.
Kempton J. W. et al., 2021 [13]	(Pará) Munduruku/31 15 mothers 16 children	NI	7.11	NI	62% MeHg ≥ 6 18.8 MeHg > 6.0	Gross motor retardation and anemia
Perini J. A. et al., 2021 [14]	(Pará) Munduruku/102 Under 15 years old	7.0 ± 4.5 (1.4–23.9)	NI	NI	49% MeHg ≥ 6	ALAD polymorphism (2/102 children). Neurological disorders.
Silva M. C. et al., 2023 [29]	(Pará) Munduruku /107 Sawré Muybu Poxo Muybu Sawré Aboy	NI	NI	8.5 ± 4.3 6.9 ± 3.5 7.4 ± 2.3 13.5 ± 4.6	NI	GSTP1 SNP (MAF) minor allele frequency G 57% 21% 15% 36.8% (total)
Jacques A. D. et al., 2024 [30]	(Roraima) Yanomami/120	3.30 (0.16 ± 10.20)	NI	NI	NI	Negative Association: Lower TIQ score; increased age; Hg levels; fish consumption
Rebouças B. H. et al., 2024 [31]	(Roraima) Yanomami/154	NI	NI	NI	3.9 ± 1.7	Chronic exposure to HG can lead to significant neurotoxicity

NI: not-investigated mercury exposure.

**Table 2:** The risk-of-bias assessment of the studies included in the article was conducted based on the criteria established by the JBI.

Study	Inclusion Criteria	Sampling	Exposure and Outcome Measures	Control of Confounding Factors	Statistical Methods Used	Bias Classification
Campos et al. (2002) [27]	Clear criteria but small sample; unclear criteria	Small and non-representative sample	Adequate hair samples but limited focus	No control for important confounding factors	Correlation analysis without multivariate adjustment	High
Dórea et al. (2005) [23]	Clear criteria but non-probabilistic sampling	Small sample (249 Munduruku, 47 Kayabi)	Reliable mercury measurements but no multivariate evaluation	No explicit control for confounding factors	Pearson correlation; lack of multivariate analysis	Moderate
Achatz et al. (2021) [5]	Clear criteria for the Munduruku population	Limited sample of 109 participants	Adequate hair samples; health evaluation subjective	No confounding control	Appropriate Poisson regression	Moderate
Barbosa et al. (1998) [26]	Clear criteria focused on indigenous women and children	Limited representation; small sample size	Reliable mercury measurement methodology	No control for dietary and other confounding factors	Adequate variance testing	Moderate
Klautau-Guimarães et al. (2005) [24]	Clear inclusion criteria for Kayabi and Munduruku	Adequate sampling of indigenous populations	Reliable mercury measures and genetic evaluation	Partial control; environmental factors not extensively covered	ANOVA and chi-square used appropriately	Moderate
Jacques et al. (2024) [30]	Clear criteria focused on Yanomami children	Representative sampling from eight villages	Reliable mercury measurements; cultural limitations in cognition assessment	Partial control for age and fish consumption	Robust analysis of mercury levels and IQ	Moderate
Vega et al. (2018) [15]	Clear criteria but limited availability of participants	Representative sampling across various villages	Reliable mercury measurements; direct measurement methods	No adequate control of confounding factors	Appropriate Poisson and Kruskal–Wallis analysis	Moderate
Oliveira et al. (2021) [7]	Well-established criteria	Small sample from three villages	Reliable hair sample measurements	No explicit control of confounding factors	Kruskal–Wallis test adequate but limited by sample size	Moderate
Kempton et al. (2021) [13]	Well-defined criteria focusing on women and children	Small sample size; no probabilistic sampling	Reliable mercury measures; limited sample	No adequate control of confounding factors	Wilcoxon and Kruskal–Wallis adequate but limited power	Moderate
Santos et al. (2002) [25]	Clear criteria but no probabilistic sampling	Small sample; limited representativeness	Reliable hair samples; limited health evaluation focus	No control of important confounding factors	Kruskal–Wallis and Spearman analysis appropriate	Moderate
Basta et al. (2021) [3]	Well-defined criteria focusing on five regions	Representative sample from various fish species	Robust mercury measurements	Adequate control of age and type of fish	Well-conducted toxicological risk analysis	Low
Perini et al. (2021) [14]	Clear criteria focusing on children under 15	Representative sample; no refusals	Accurate mercury measurements and genotyping	Adequate control for age and fish consumption	Genetic analyses and associations with clinical outcomes	Low
Rebouças et al. (2024) [31]	Clear and comprehensive criteria	Adequate and representative sample	Robust hair sample measurements	Control of confounding factors like age and health	Robust analysis with Poisson and ROC	Low
Silva et al. (2023) [29]	Clear criteria for Indigenous Munduruku	Specific sampling; no refusals	Reliable mercury and GSTP1 assessments	Partial control for age; dietary factors not discussed	Logistic regression analysis used appropriately	Low

**Table 3:** Acute and chronic symptoms of mercury exposure in adults and children and fetal risks.

Type of Exposure	Symptoms in Adults	Symptoms in Children	Fetal Risks
Acute Exposure	Tremors	Irritability	Premature birth
	Headache	Concentration difficulties	Low birth weight
	Nausea and vomiting	Skin rashes	Fetal death
	Dizziness	Loss of appetite	
	Respiratory difficulty		
	Gastrointestinal disorders		
Chronic Exposure	Cognitive impairments	Cognitive development deficits	Congenital malformations
	Peripheral neuropathy	Motor delays	Delayed neuropsychomotor development
	Memory loss	Learning difficulties	Permanent cognitive disabilities
	Motor difficulties	Persistent neurological deficiencies	Risk of cerebral palsy
	Visual and auditory changes		
	Chronic fatigue		
